# The value of multiparameter combinations for predicting difficult airways by ultrasound

**DOI:** 10.1186/s12871-022-01840-0

**Published:** 2022-10-05

**Authors:** Jianling Xu, Bin Wang, Mingfang Wang, Weidong Yao, Yongquan Chen

**Affiliations:** Department of Anesthesiology, Yijishan Hospital, the First Affiliated Hospital of Wannan Medical College, 2 Zheshanxi ST, Wuhu, 241001 China

**Keywords:** Airway ultrasonography, Difficult intubation, General anesthesia, Hyomental distance, Tongue thickness, Mandibular condylar mobility

## Abstract

**Background:**

Based on the upper airway anatomy and joint function parameters examined by ultrasound, a multiparameter ultrasound model for difficult airway assessment (ultrasound model) was established, and we evaluated its ability to predict difficult airways.

**Methods:**

A prospective case-cohort study of difficult airway prediction in adult patients undergoing elective surgery with endotracheal intubation under general anesthesia, and ultrasound phantom examination for difficult airway assessment before anesthesia, including hyomental distance, tongue thickness, mandibular condylar mobility, mouth opening, thyromental distance, and modified Mallampati tests, was performed. Receiver operating characteristic (ROC) curve analysis was used to evaluate the effectiveness of the ultrasound model and conventional airway assessment methods in predicting difficult airways.

**Results:**

We successfully enrolled 1000 patients, including 51 with difficult laryngoscopy (DL) and 26 with difficult tracheal intubation (DTI). The area under the ROC curve (AUC) for the ultrasound model to predict DL was 0.84 (95% confidence interval [CI]: 0.82–0.87), and the sensitivity and specificity were 0.75 (95% CI: 0.60–0.86) and 0.82 (95% CI: 0.79–0.84), respectively. The AUC for predicting DTI was 0.89 (95% CI: 0.87–0.91), and the sensitivity and specificity were 0.85 (95% CI: 0.65–0.96) and 0.81 (95% CI: 0.78–0.83), respectively. Compared with mouth opening, thyromental distance, and modified Mallampati tests, the ultrasound model predicted a greater AUC for DL (*P* < 0.05). Compared with mouth opening and modified Mallampati tests, the ultrasound model predicted a greater AUC for DTI (*P* < 0.05).

**Conclusions:**

The ultrasound model has good predictive performance for difficult airways.

**Trial registration:**

This study is registered on chictr.org.cn (ChiCTR-ROC-17013258); principal investigator: Jianling Xu; registration date: 06/11/2017).

**Supplementary Information:**

The online version contains supplementary material available at 10.1186/s12871-022-01840-0.

## Introduction

The assessment and prediction of a difficult airway is the first step in airway management. Failure to identify a difficult airway can lead to life-threatening complications, such as brain injury and death [1]. Research shows that predicting difficult airways is challenging, and intubation is difficult in only 1 in 4 patients with difficult airways which are expected [2]. At present, the commonly used clinical indicators for predicting difficult airways, such as mouth opening, thyromental distance, modified Mallampati tests, and neck mobility, have unreliable prediction effects and poor sensitivity and specificity [3, 4, 5]. The formation of a difficult airway is related to the internal anatomy of the upper airway, and ultrasonography is a very convenient method used to assess the internal anatomy of the upper airway. Ultrasound is important for airway management. Studies have shown that ultrasound measurements of tongue thickness [6], hyomental distance [7], and mandibular condylar mobility [8, 9] are valuable in predicting difficult airways. However, difficult airway is not determined by a single anatomical factor, and the sensitivity and specificity of a single index for airway prediction are not ideal. The purpose of this study was to combine multiple parameters based on ultrasound measurements to establish a comprehensive ultrasound assessment method (ultrasound model) for the prediction of difficult airways and to compare the model with commonly used airway assessment methods to evaluate the effectiveness of the ultrasound model in predicting difficult airways.

## Methods

This prospective case-cohort study was performed following the tenets of the Declaration of Helsinki. Approval was obtained from the Ethics Committee of Yijishan Hospital of Wanan Medical College (NO: (2019) 89). The study subjects were patients hospitalized at Yizhishan Hospital from September 2019 to June 2020. Written informed consent was obtained from all patients for the publication of their data. The study protocol was registered at chictr.org.cn (ChiCTR-ROC-17013258). This study adheres to the applicable STROBE protocol.

### Establishment of the ultrasound model

Based on the previous work of our team [6, 8, 10], it has been clinically verified by a large sample that tongue hypertrophy independently predicts difficult airways. A tongue thickness > 61 mm can indicate a difficult airway [6]. Ultrasound measurement of the mandibular condylar mobility was found to be an independent risk factor for DL, and the best predictive performance was when the mandibular condylar mobility was ≤ 10 mm, the sensitivity and specificity were 0.81 (0.60–0.95) and 0.91 (0.87–0.94), respectively, and the AUC and its 99% CI were 0.93 (99% CI 0.90–0.96) [8]. Some studies have confirmed [10, 11, 12] that the measurement of the hyomental distance by ultrasound can accurately predict difficult airways, and a hyomental distance ≤ 51 mm is used as a positive criterion for predicting difficult airways [10]. Based on the previous work of our team and with respect to related literature, this study combines multiple parameters based on ultrasonic measurements to establish a new ultrasonic model, including tongue thickness > 61 mm, mandibular condylar mobility ≤ 10 mm, and hyomental distance ≤ 51 mm. According to the cutoff values, positive data were assigned 1 point, and the total score was 3 points. The effect of a new ultrasound model for predicting difficult airways was evaluated.

### Ultrasonic positioning measurement method

#### Tongue thickness and hyomental distance

**Fig. 1:**
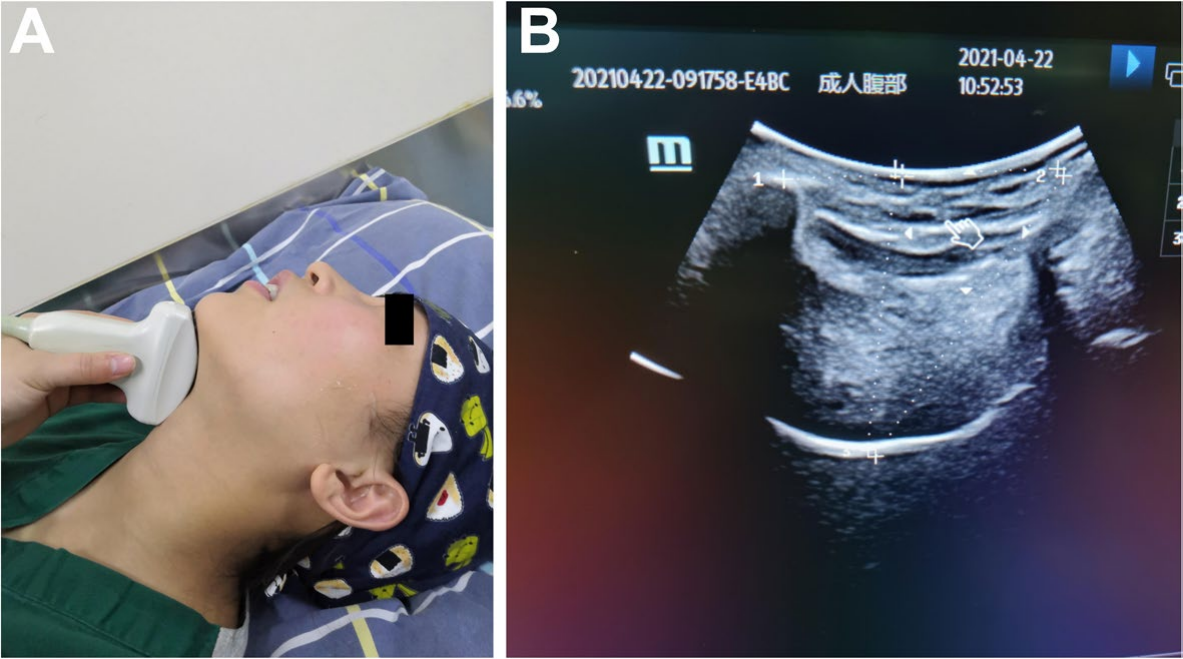
The method of measuring the hyomental distance and tongue thickness under ultrasound. The body surface position of the ultrasound probe (**A**) and imaging (**B**). D12 represents the hyomental distance, which is 52.31 mm. D34 represents the tongue thickness, which is 46.83 mm

Using a convex array low-frequency probe placed in the midsagittal plane of the patient’s neck, one end of the probe is flat against the top of the mandible, and the other end points to the sternum end; the probe is perpendicular to the surface of the neck. The sound shadow of the mandible, hyoid, and tongue can be displayed on the ultrasound screen. Tongue thickness and hyoid-mental distance are measured by ultrasound imaging [6, 9] (Fig. 1). 

#### Mandibular condylar mobility

**Fig. 2:**
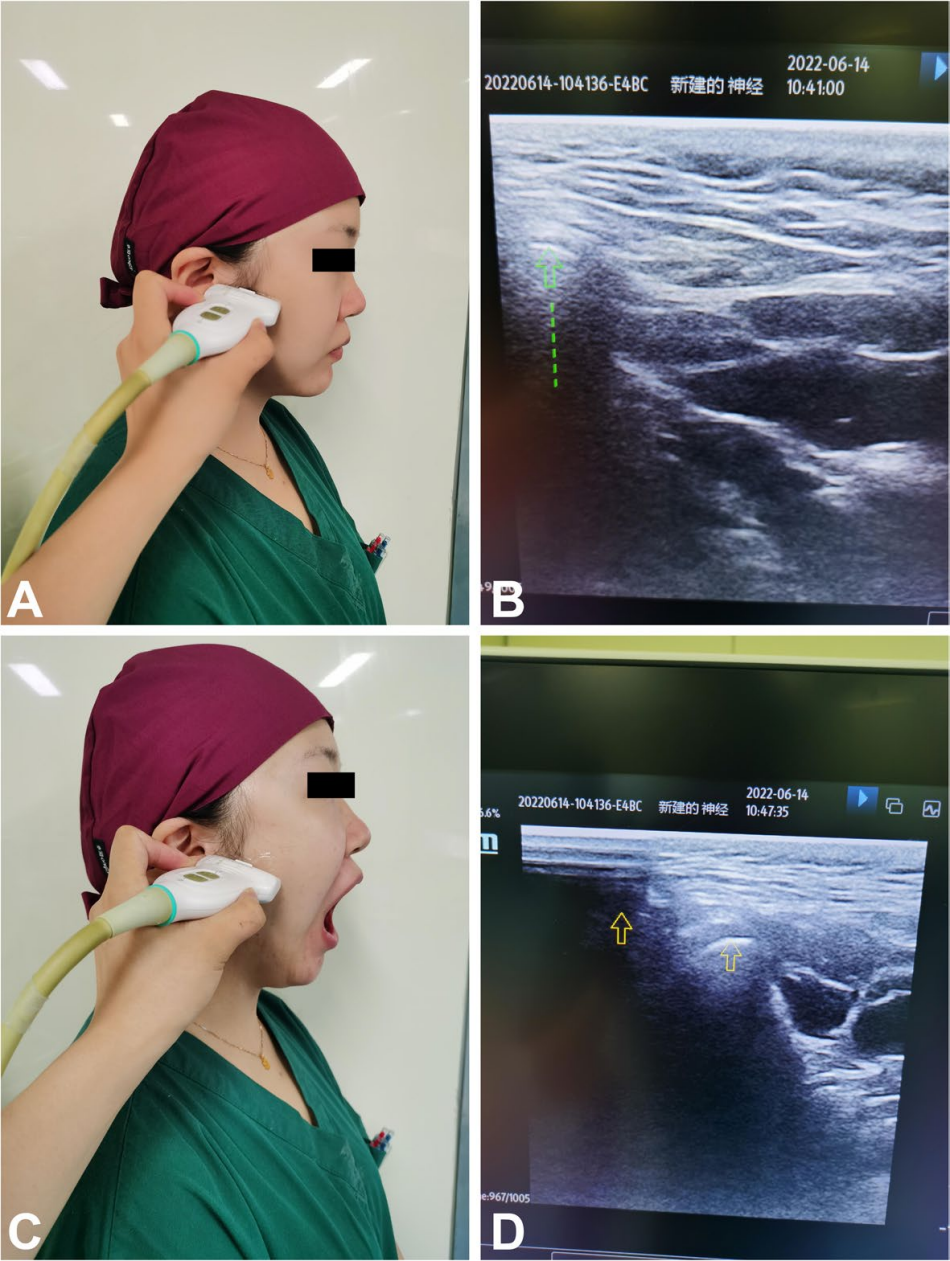
Temporomandibular joint mobility sonographic methods and condylar translation measurement. Ultrasound probe position (**A** and **C**) and images (**B** and **D**) captured separately when the mouth was opened and closed. When the 2 images were compared, the mandibular condyle position was shifted from one point (crosshair marked “1” in **B** and **D**) to the other (the other crosshair in **D**). The mandibular condylar mobility is 11.10 mm (D12)

With the patient in the supine position and the head and neck in a neutral position, the linear array high-frequency probe is placed in front of the patient’s ear, and the two ends of the probe point to the patient’s external auditory canal and the tip of the nose. The angle is adjusted so that the ultrasound probe is perpendicular to the skin, and the mandibular condyle can be displayed on the ultrasound screen. Without moving the probe position, the patient is allowed to open their mouth to the maximum extent, and the distance between the open mouth and the closed mouth after freezing the image is measured, which is the moving distance of the maxillary condyle [8] (Fig. 2).

### Patient recruitment

Patients who were scheduled for elective surgery with endotracheal intubation under general anesthesia were recruited and signed informed consent forms. The inclusion criteria were adult males and females (aged 18 to 90 years) with an ASA physical status of I, II, or III. The exclusion criteria were upper airway anatomical deformity, trauma or tumor, subglottic stenosis, established difficult airway requiring awake endotracheal intubation, or incomplete or missing data.

Several variables were measured by the research team before the induction of anesthesia. The first metric was modified Mallampati tests. The patient was required to open mouth. In grade I, the soft palate, pharyngeal and palatine arch, and uvula can be seen. In grade II, the soft palate, pharyngeal and palatine arch, and uvula are partially covered by the tongue. In grade III, only the soft palate can be seen. In grade IV, none of them are visible. Grades III and IV indicate DL [13]. The second variable was mouth opening. The patient was required to open mouth to the maximum extent, and the distance between the incisal edges of the upper and lower central incisors was measured to determine if the distance was less than 3 cm, which was considered DTI [13]. The third metric was thyromental distance, which is the distance from the thyroid cartilage notch to the tip of the mandible when the patient’s head is extended; a distance less than 6.5 cm is considered difficult to intubate with a laryngoscope [13]. Before the induction of anesthesia, tongue thickness, hyomental distance, and mandibular condylar mobility were measured by an anesthesiologist who was proficient in ultrasonic measurement methods using an ultrasonic examination instrument (Navis type, Shenzhen Watson, China).

### Anesthesia and endotracheal intubation

After the patient enters the operating room, the peripheral vein is opened, and the patient’s blood pressure, electrocardiogram, and pulse oximetry are monitored. Anesthesia induction involved the following: midazolam 0.05 mg/kg, sufentanil 0.4–0.6 μg/kg, propofol 1–2 mg/kg, and rocuronium 0.6 mg/kg. After three minutes, an experienced attending anesthesiologist used a common laryngoscope to expose the glottis for tracheal intubation, and the endotracheal tube model was selected by the intubating physician based on experience. The intubating physician is not informed of the airway measurement data, but the airway can be assessed empirically. If intubation fails, then mask ventilation is used to increase the patient’s pulse oxygen saturation above 98%, and the procedure is repeated. Glottic exposure is assessed according to the Cormack-Lehane (C-L) scale [14]. In grade I, most of the glottis can be seen. In grade II, only arytenoid cartilage can be seen. In grade III, only the epiglottis can be seen, and in grade IV, neither the glottis nor the epiglottis can be seen. Grade III or IV is associated with difficult laryngeal exposure.

### Observation end point

The primary outcome was DTI refers to the anesthesiologist’s experience in using an ordinary laryngoscope for tracheal intubation; a DTI requires more than 10 min, more than three attempts or the need to replace high-level intubation equipment (such as visual equipment, light rods, etc.).

The secondary observation indicator was DL, referring to CL grades III and IV.

When a difficult airway is suspected or encountered, the difficult airway team is immediately called for support. According to the specific situation, a video laryngoscope, a fiberoptic bronchoscope, light-rod guided tracheal intubation, or emergency establishment of a surgical airway is used. The guidelines for difficult airway management [15] are followed to maximize patient safety.

### Statistical analysis

SPSS 18.0 and MedCalc 12.7 statistical software were used for data analysis. Normally distributed measurement data are expressed as the mean ± standard deviation, and categorical or graded data are expressed as the frequency or rate. Comparisons between groups were performed using the t test, X2 test, or rank-sum test according to the situation. Logistic regression analysis and ROC curve analysis were used to evaluate the performance of ultrasound models and other methods for predicting difficult airways and to calculate sensitivity and specificity. For two-sided tests, *P* < 0.05 was considered a statistically significant difference. Based on that the type I error rate of the set test was 0.05, the type II error rate was 0.20, the input AUC value was 0.9, the null hypothesis value was 0.8, the negative-to-positive ratio was 19, and the calculated sample size was 1000 cases.

## Results

**Fig. 3:**
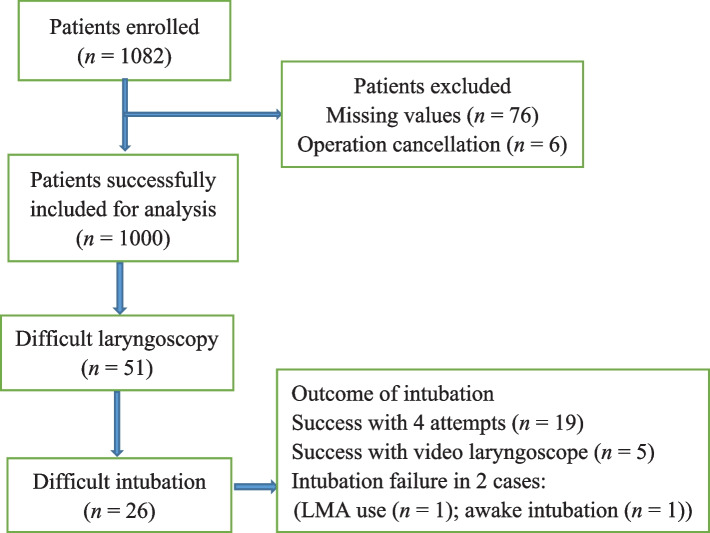
Study flow chart and patient outcomes

A total of 1082 patients were enrolled. Seventy-six patients had incomplete data, and six patients were excluded before anesthesia induction. Ultimately, 1000 patients were included. Figure 3 shows the outcomes of the patients. Fifty-one (5.1%) patients had DL, 26 (2.6%) patients had DTI.

The results of the comparison of variables between the difficult airway group and the non-DA group are shown in Table 1. There was no significant difference in height or weight between the groups, but there were significant differences in age, mouth opening, thyromental distance, modified Mallampati tests, and ultrasound assessment method between the two groups (*P* < 0.05).

**Table 1:** Comparison of patient general information and measured variables

Variable and outcome	DTI (*n* = 26)	Non-DTI (*n* = 974)	*P* value	DL (*n* = 51)	Non-DL (*n* = 949)	*P* value
Sex (male/female)/n	15/11	504/470		33/18	486/463	-
Age (years)	57.77 ± 11.51	51.15 ± 14.12	*P* = 0.18	58.58 ± 12.41	50.92 ± 14.08	*P* < 0.05
Height (cm)	164.77 ± 7.15	164.54 ± 7.36	0.88	165.28 ± 7.05	164.50 ± 7.37	*P* = 0.47
Weight (kg)	62.40 ± 10.03	63.21 ± 10.65	0.70	62.29 ± 11.36	63.23 ± 10.60	*P* = 0.54
Body mass index (kg (m^2^)^−1^)	22.99 ± 3.33	23.28 ± 3.29	0.65	22.77 ± 3.71	23.30 ± 3.27	*P* = 0.27
Mouth opening (cm)	33.27 ± 6.87	40.21 ± 5.22	*P* < 0.05	34.52 ± 6.08	40.31 ± 5.18	*P* < 0.05
Modified Mallampati tests	3.08 ± 0.56	2.18 ± 0.85	*P* < 0.05	2.88 ± 0.77	2.17 ± 0.85	*P* < 0.05
Thyromental distance (cm)	66.73 ± 7.80	78.20 ± 8.15	*P* < 0.05	69.74 ± 8.50	78.34 ± 8.10	*P* < 0.05
Ultrasound model	2.35 ± 0.75	0.84 ± 0.82	*P* < 0.05	0.82 ± 0.81	2.08 ± 0.82	*P* < 0.05

**Fig. 4:**
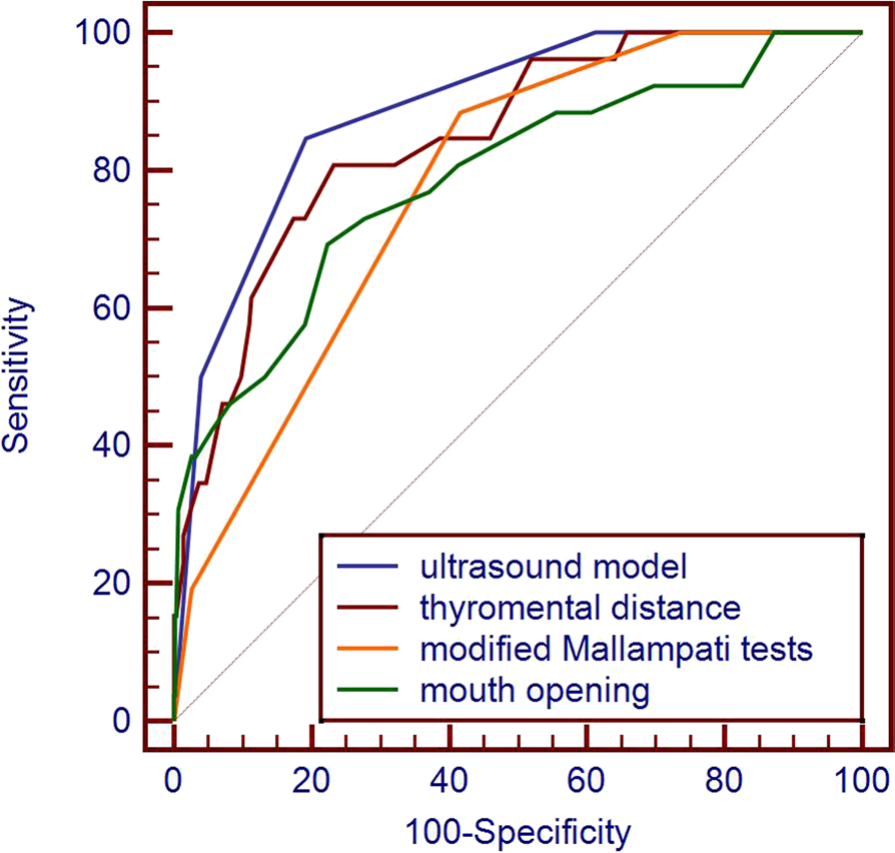
The ROC curve of the ultrasound assessment method, modified Mallampati tests, thyromental distance, and mouth opening to predict difficult tracheal intubation

The AUCs of the ultrasound assessment method, modified Mallampati test, nail-mental distance, and mouth opening to predict DTI were 0.89 (0.87–0.91), 0.78 (0.75–0.80), 0.85 (0.82–0.870), and 0.79 (0.76–0.81), respectively. Compared with the modified Mallampati tests and mouth opening, the AUC of the ultrasound assessment method was increased (*P* < 0.05), as shown in Table 2. The ROC curves of the ultrasound assessment method, modified Mallampati tests, thyromental distance, and mouth opening to predict DTI are shown in Fig. 4.

**Table 2:** Significance analysis of each parameter for the prediction of DTI

Parameters and cutoff value	Sensitivity (95% CI)	Specificity (95% CI)	PPV (95% CI)	NPV (95% CI)	AUC (95% CI)
Modified Mallampati tests (> 2)	0.88 (0.70–0.98)	0.58 (0.55–0.61)	0.05 (0.05–0.06)	1.00 (0.99–1.00)	0.78 (0.75–0.80)^a^
Thyromental distance (< 6.5 cm)	0.46 (0.27–0.67)	0.93 (0.91–0.94)	0.15 (0.10–0.22)	0.99 (0.98–0.99)	0.85 (0.82–0.870
Mouth opening (< 3 cm)	0.38 (0.20–0.59)	0.97 (0.96–0.98)	0.28 (0.17–0.42)	0.98 (0.98–0.99)	0.79 (0.76–0.81) ^a^
Mandibular condylar mobility (≤ 10 mm)	0.81 (0.61–0.93)	0.80 (0.77–0.82)	0.10 (0.08–0.12)	0.99 (0.99–1.00)	0.87 (0.85–0.89)
Hyomental distance (≤ 51 mm)	0.77 (0.56–0.91)	0.75 (0.72–0.78)	0.08 (0.06–0.09)	0.99 (0.98–1.00)	0.86 (0.84–0.88)
Tongue thickness (> 61 mm)	0.78 (0.56–0.91)	0.61 (0.58–0.64)	0.05 (0.04–0.06)	0.99 (0.98–1.00)	0.76 (0.74–0.79)
Ultrasound model (> 1)	0.85 (0.65–0.96)	0.81 (0.78–0.83)	0.11 (0.09–0.13)	1.00 (0.99–1.00)	0.89 (0.87–0.91)

Compared with the ultrasound comprehensive evaluation method

*CI* Confidence interval, *PPV* Positive predictive value, *NPV* Negative predictive value

^a^*P* < 0.05

**Fig. 5:**
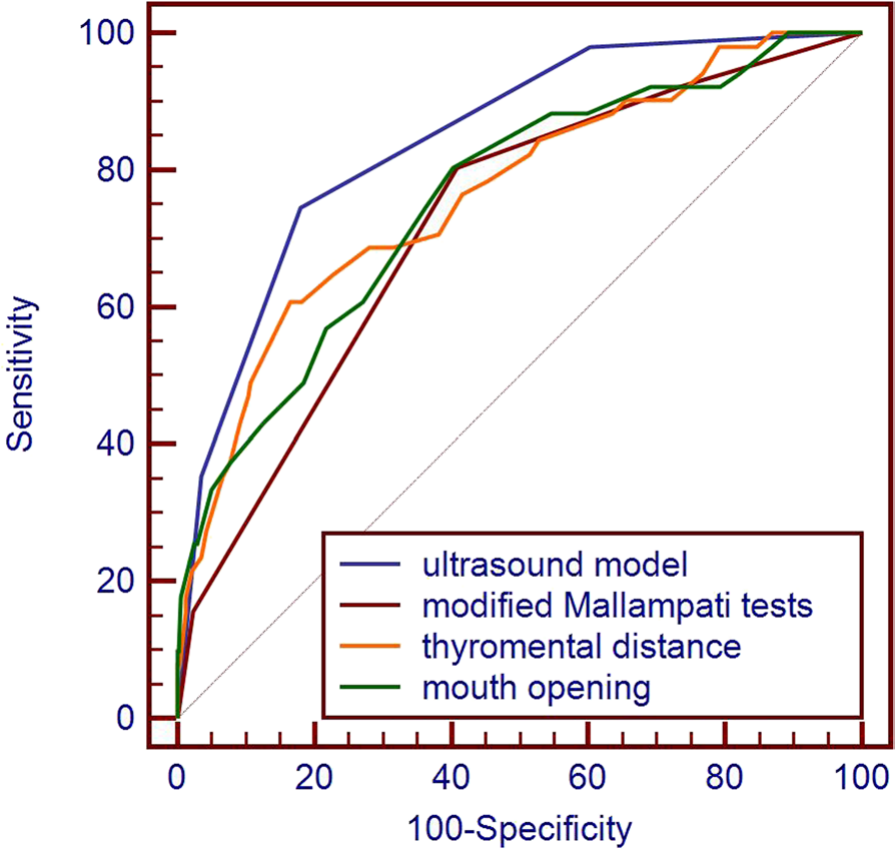
The ROC curve of the ultrasound assessment method, modified Mallampati tests, thyromental distance, and mouth opening to predict difficult laryngoscopy

The AUCs of the ultrasound assessment method, modified Mallampati test, thyromental distance, and mouth opening to predict DL were 0.84 (0.82–0.87), 0.72 (0.69–0.75), 0.77 (0.74–0.79), and 0.76 (0.73–0.78), respectively.Compared with the modified Mallampati tests, thyromental distance, and mouth opening, the AUC of the ultrasound assessment method was increased (*P* < 0.05), as shown in Table 3. The ROC curves of the ultrasound assessment method, modified Mallampati tests, thyromental distance, and mouth opening to predict difficult laryngoscopy are shown in Fig. 5.

**Table 3:** Significance analysis of each parameter for the prediction of DL

Parameters and cutoff value	Sensitivity (95% CI)	Specificity (95% CI)	PPV (95% CI)	NPV (95% CI)	AUC (95% CI)
Modified Mallampati tests (> 2)	0.80 (0.67–0.90)	0.59 (0.56–0.62)	0.10 (0.08–0.11)	0.98 (0.97–0.99)	0.72 (0.69–0.75) ^a^
Thyromental distance (< 6.5 cm)	0.35 (0.22–0.50)	0.93 (0.92–0.95)	0.22 (0.16–0.31)	0.96 (0.96–0.97)	0.77 (0.74–0.79) ^a^
Mouth opening (< 3 cm)	0.25 (0.14–0.40)	0.98 (0.96–0.99)	0.36 (0.23–0.51)	0.96 (0.95–0.97)	0.76 (0.73–0.78) ^a^
Mandibular condylar mobility (≤ 10 mm)	0.76 (0.63–0.87)	0.81 (0.78–0.84)	0.18 (0.15–0.21)	0.99 (0.98–0.99)	0.84 (0.82–0.87)
Hyomental distance (≤ 51 mm)	0.67 (0.52–0.79)	0.75 (0.72–0.78)	0.13 (0.10–0.15)	0.98 (0.97–0.98)	0.79 (0.76–0.81)
Tongue thickness (> 61 mm)	0.65 (0.50–0.78)	0.61 (0.58–0.64)	0.08 (0.07–0.10)	0.97 (0.96–0.98)	0.69 (0.66–0.71)
Ultrasound model (> 1)	0.75 (0.60–0.86)	0.82 (0.79–0.84)	0.18 (0.15–0.22)	0.98 (0.97–0.99)	0.84 (0.82–0.87)

Compared with the ultrasound comprehensive evaluation method

*CI* Confidence interval, *PPV* Positive predictive value, *NPV* Negative predictive value

^a^*P* < 0.05

The ultrasound assessment method determined by the Youden index had a cutoff value of > 1 point for predicting DTI and DL. The AUC of the ultrasound assessment method for predicting DTI was 0.89 (0.87–0.91), and the sensitivity and specificity were 0.85 and 0.81, respectively. The ultrasound assessment method predicted DL with an AUC of 0.84 (0.82–0.87) and a sensitivity and specificity of 0.75 and 0.82, respectively.

## Discussion

Our study shows that, The AUC of the ultrasound assessment method for predicting DTI and DL was 0.89 and 0.84, respectively. The sensitivity and specificity are also high. It is suggested that the ultrasound assessment method is effective in predicting DTI and DL and can effectively predict difficult preoperative airways. These findings are consistent with previous research reports [6, 8, 10]. Even though the continuous measurement variables were converted into dichotomous variables, which would reduce the AUC but could increase the convenience of clinical application.

Ultrasound can be used to identify anatomical landmarks of the upper airway and for accurate measurements. Carsetti et al. [16] pointed out that upper airway ultrasound may be a powerful tool for improving the performance of difficult airway management predictive tests, providing an objective assessment of specific index tests and thus restricting the interobserver variability. The current guidelines for preprocedural evaluation recommend using a combination of the validated tests to predict, and thereby, manage difficult airways, because no factor can provide an accurate prediction when assessed alone. [17]. The integration of ultrasound airway assessment with routinely used tests should be investigated to clarify the potential role of this technique in periprocedural patient evaluation [18].

The mandibular condyle is a motion joint that can change or shift with the insertion of a laryngoscope. The widely used clinical mouth opening and upper lip bite grades are indirect reflections of mandibular condyle mobility. Therefore, the mobility of the mandibular condyle must be the main factor involved in determining a difficult airway [8]. Ho et al. [19] found that ultrasound can quickly and accurately assess mandibular condylar mobility and is a repeatable operation method, pointing out that mandibular condylar mobility is linearly related to the degree of mouth opening. The hyomental distance can directly reflect whether the patient’s mandibular space is sufficient and is an effective indicator for predicting difficult airways [20, 21]. Ultrasound can accurately locate the hyoid bone and measure the hyomental distance, thereby improving the prediction of difficult airways [22]. Tongue hypertrophy can interfere with laryngeal exposure, thus leading to difficult intubation. Yao et al. [6] studied 2254 patients and measured tongue thickness by ultrasound. They found that tongue hypertrophy was an independent factor for predicting difficult airway and pointed out that tongue thickness > 6.1 cm could indicate a difficult airway. The results of this study are similar to this finding.

We selected mandibular condylar mobility, the hyomental distance, and tongue thickness to demonstrate the ability of individual parameters and combinations of parameters based on upper airway ultrasonography to predict difficult airways. Agarwal et al. [22] studied the performance of the ultrasonic measurement of multiple airway parameters in predicting difficult airways and proved that ultrasonic comprehensive prediction methods can effectively predict difficult airways.

The use of point-of-care ultrasound (POCUS) has been limited given the lack of portability and the substantial cost of larger, traditional ultrasound machines. With the advent of newer, compact, handheld devices, the use of ultrasound in guiding airway management has become more feasible. In addition to greater mobility, the image quality and analytic features of newer handheld POCUS devices have improved when compared to earlier systems [23]. Upper airway POCUS has the potential to become the first-line noninvasive adjunct assessment tool in airway management [24].

The purpose of our study was to observe the ability of ultrasound to measure upper airway anatomical parameters in addition to predicting DTI and DL. Commonly used laryngoscopes can expose the glottis and determine C-L grading. Therefore, we chose commonly used laryngoscopes as the intubation devices. A video laryngoscope was used as a rescue device in patients with intubation failure.

There are some other limitations in the present study. First, the included research subjects were only elective patients with endotracheal intubation under general anesthesia. Teenagers and patients with facial anatomical deformities were excluded. Second, this was a single-center study; that is, all the selected subjects were from the same hospital.

In summary, compared with modified Mallampati tests, hyomental distance, and mouth opening, the preoperative ultrasound comprehensive prediction model to evaluate difficult airways has a better predictive effect.

## Supplementary Information

**Additional file 1.**

## Data Availability

The datasets generated and analyzed during the current study are not publicly available due to institutional restrictions but are available from the corresponding author on reasonable request.
